# Low Cognitive Impulsivity Is Associated with Better Gain and Loss Learning in a Probabilistic Decision-Making Task

**DOI:** 10.3389/fpsyg.2017.00204

**Published:** 2017-02-16

**Authors:** Pablo Cáceres, René San Martín

**Affiliations:** ^1^Facultad de Economía y Empresa, Centro de Neuroeconomía, Universidad Diego PortalesSantiago, Chile; ^2^Center for Cognitive Neuroscience, Duke UniversityDurham, NC, USA

**Keywords:** cognitive impulsivity, reward-based learning, economic decision-making, cognitive control

## Abstract

Many advances have been made over the last decades in describing, on the one hand, the link between reward-based learning and decision-making, and on the other hand, the link between impulsivity and decision-making. However, the association between reward-based learning and impulsivity remains poorly understood. In this study, we evaluated the association between individual differences in loss-minimizing and gain-maximizing behavior in a learning-based probabilistic decision-making task and individual differences in cognitive impulsivity. We found that low cognitive impulsivity was associated both with a better performance minimizing losses and maximizing gains during the task. These associations remained significant after controlling for mathematical skills and gender as potential confounders. We discuss potential mechanisms through which cognitive impulsivity might interact with reward-based learning and decision-making.

## Introduction

Recent advances in understanding the neurocognitive basis of decision-making have emphasized the key role of both reinforcement learning and impulsivity. On the one hand, computational theories of reinforcement learning have provided a fruitful framework to describe and predict adaptive decision-making on the basis of reward-based learning mechanism (Sutton and Barto, 1998; Ridderinkhof et al., 2004; Heekeren et al., 2007; Dayan and Niv, 2008; Eppinger et al., 2011; Lee et al., 2012). On the other hand, impulsivity has been consistently linked to suboptimal and maladaptive decision-making in healthy and clinical populations (Bechara, 2005; Franken et al., 2008; Dalley et al., 2011; Kim and Lee, 2011; Ottaviani and Vandone, 2011). Despite these recent advances in the understanding of the functional link between decision-making and reward-based learning and impulsivity, little is still known about the relationship between impulsivity and reward-based learning, since most part of the research on such processes has been made as separate lines of inquiry. Here, we contribute to fill such gap by examining the relationship between cognitive impulsivity and reward-based learning during a probabilistic decision-making task.

Reinforcement learning is a general framework for the study of the way in which natural and artificial systems optimize their behavior by learning to predict the consequences of their actions in the environment. As such, reinforcement learning provides and important theoretical perspective for understanding and modeling adaptive decision-making processes in both in humans and in non-human systems (Dayan and Niv, 2008; Lee et al., 2012). The reinforcement learning literature has distinguished between learning from negative feedback (i.e., loss-learning) and learning from positive feedback (i.e., gain-learning; Kuhnen and Knutson, 2005; Samanez-Larkin et al., 2010; Voon et al., 2010; Cavanagh et al., 2011; Knutson et al., 2011; San Martín et al., 2013; Kuhnen, 2015; Kuhnen and Miu, 2015). In laboratory settings these systematic propensities to seek gains and to avoid losses have been studied using the concepts of *gain-maximization* and *loss-minimization*, where gain-maximization refers to the ability of obtaining the best gain in a gain scenario, and loss-minimization refers to the ability of avoiding the worse loss in a loss scenario (Venkatraman et al., 2011; San Martín et al., 2013, 2014).

In a parallel line of research, impulsivity has arisen as a key ingredient of neurocognitive models of decision-making, underscoring the deleterious effect of high levels of impulsivity on decision-making patterns (Bechara, 2005; Dalley et al., 2011; Kim and Lee, 2011; Ottaviani and Vandone, 2011). Impulsivity is typically characterized as the tendency to act prematurely with little or no forethought and as systematic failures in suppressing inappropriate motor, cognitive or emotional responses (Durana and Barnes, 1993; Meda et al., 2009). Here, we measured impulsivity using the “cognitive reflection test” (CRT), an instrument that emphasizes the cognitive dimension of impulsivity, this is, the tendency to make rash choices without an appropriate evaluation of the alternatives. The CRT is composed of three items designed to elicit an intuitive but incorrect response (“lures”) when approaching a reasoning problem that actually requires a slow and reflexive response. Accordingly, it has been used as a measure of both cognitive reflection and cognitive impulsivity (Cokely and Kelley, 2009; Johnson et al., 2012; Baron et al., 2015; Cueva et al., 2015).

Interestingly, there are various parallels between findings linking reward-based learning and decision-making and findings linking impulsivity and decision-making. More remarkable, recent research has highlighted the role of the dopaminergic system both in reward-learning (Frank et al., 2004; Klein et al., 2007; Schönberg et al., 2007; Pizzagalli et al., 2008; Eppinger et al., 2011; Jocham et al., 2014; Cox et al., 2015) and impulsivity (Kalivas and Volkow, 2005; Dalley et al., 2007; Pattij and Vanderschuren, 2008; Besson et al., 2009; Lee et al., 2009; Eagle et al., 2011; Buckholtz et al., 2016). Specifically, low availability of dopamine D2/3-like receptors in the striatum is associated both with bad performance in reward-based learning (Klein et al., 2007; Jocham et al., 2014) and with high impulsivity (Dalley et al., 2007; Besson et al., 2009; Lee et al., 2009). Here, we directly evaluated the hypothesis that high cognitive impulsivity, as measured with the CRT, will be associates with a worse performance maximizing gains and minimizing losses in learning-based decision-making.

## Materials and methods

### Participants

Twenty-one healthy, right-handed, adult volunteers (12 women, 9 men) participated in this study (ages, 18–25 years; Mean = 21.76). Participants were financially compensated for their time ($7/h). They received an extra bonus (Mean = $3; *SD* = $1.5) which was proportional to the points earned during the experimental session. All participants signed a voluntary consent form in accordance with the Declaration of Helsinki, in addition to the approval granted by the ethical committee of Universidad Diego Portales.

### Tasks and procedures

#### Decision-making task

We used a probabilistic decision-making task has been previously used by San Martín et al. (2013, 2014) to study the event-related brain potentials associated with learning and decision-making. The primary goal of the task is to learn, by trial and error, the probabilistic association between a set of symbols and the probability of winning vs. losing on each trial, and then use that information to choose between a small and a large bet on each trial. Before data collection, subjects were told that each trial would start with the presentation of two symbols, and that some symbols tended to precede losses whereas others tended to precede gains. Participants were informed that the probabilistic relationship between symbols and outcomes would remain constant during the session, that they would win or lose points according to the amount of their bets (i.e., 8 or 2 points) in every trial, and that at the end of the session they would receive a monetary reward in proportion to the points collected in the decision-making task.

Each experimental session began with a 20-trial practice with each participant seated in front of a computer screen. For the practice sessions we used a set of symbols specifically selected to differ from the set of symbols that posteriorly were used during data collection. After the initial practice, subjects performed 600 trials over course of a single experimental session divided into 30 ~1.7 min blocks. Feedback about the cumulative performance of the participant (i.e., number of points collected) was provided after the first half and at the end of the session.

**Figure 1:**
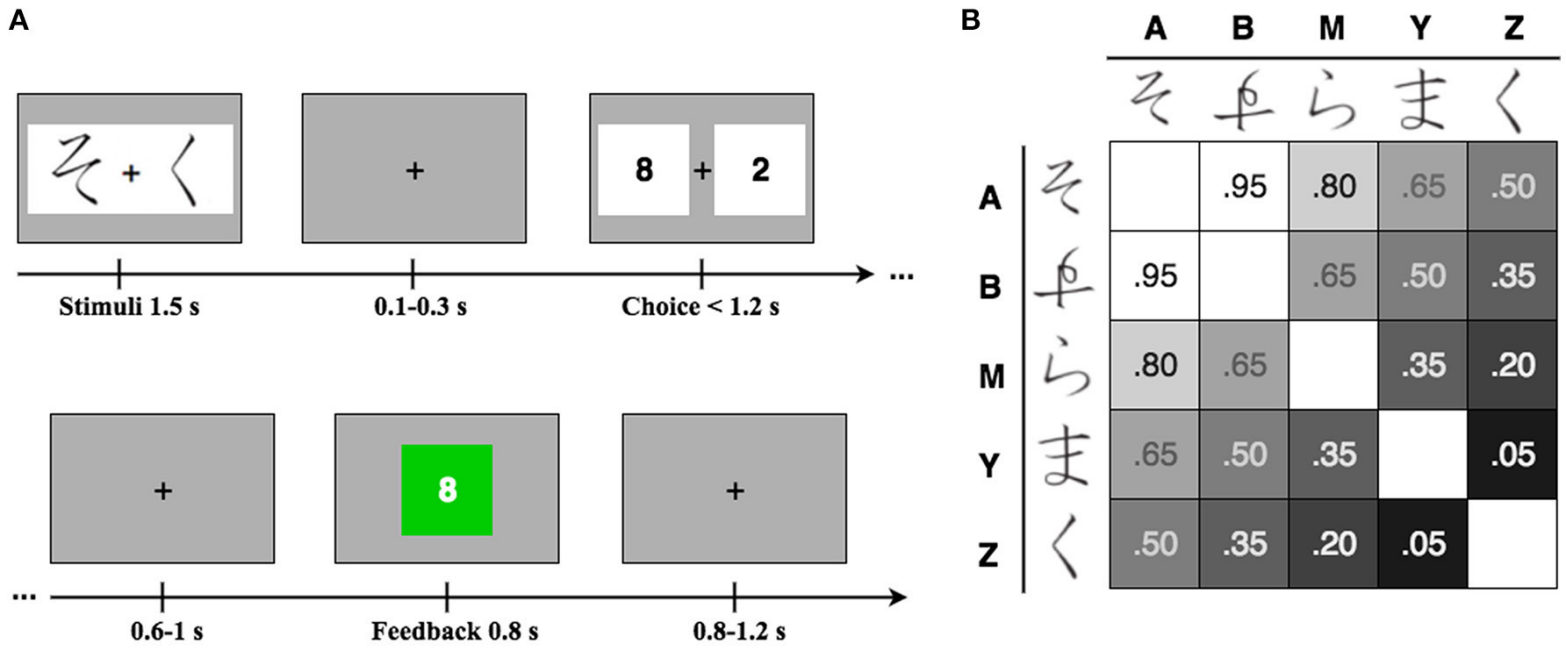
**(A)** Experimental design. On each trial subjects had to pay attention to a pair of symbols and a fixation cross in the center of the screen. Then, they had to choose their wager preference by pressing the button in a keyboard that matches the side of the screen where their corresponding preference was located. Feedback about the outcome of the bet was presented in a green box if the subject won on that trial or in a red box if the participant lost on that trial. The numeral presented in the box corresponded to the amount of points obtained in each trial, in correspondence with the wager previously selected. **(B)** Cues and win probability. Symbols were randomly selected from a set of 20 different pairs of “Hiragana” characters, without replacement. The panel displays all-possible combinations of symbols and their corresponding associated probability of winning vs. losing on each trial.

The temporal deployment of the task is represented in the Figure 1A. Each trial started with the presentation of a pair of symbols and a fixation cross in the center of the screen for 1500 ms. The symbols were randomly selected, without replacement, from a set of 20 unique pairs of Hiragana characters. Figure 1B display all-possible combinations of symbols and their corresponding associated probability of winning or losing in each trial. After an interstimulus interval (ISI) jittered between 100 and 300 ms, the numerals “8” and “2” were displayed as wager alternatives, which were randomly selected to appear at the left or at the right of a fixation cross. Subjects choose their wager preference by pressing a keyboard button matching the location of the chosen alternative in the screen. Feedback about the outcome of the trial was presented after an ISI jittered between 600 and 1000 ms. The wagered points appeared in a green box if the subject won on that trial or in a red box if the participant lost on that trial. If no response was selected within 1200 ms after the presentation of the wager alternatives, a “no response” message and a box showing a lost of eight points was displayed. The following trial started after an intertrial interval jittered between 800 and 1200 ms.

The outcome of the bet (i.e., winning or losing the wagered points) was probabilistically determined according to the probability of winning [p(win)] associated with the pair of symbols presented on each particular trial (Figure 1B). The p(win) associated with each pair of symbols corresponded to an adjustment from 50% according to the increment or decrement of p(win) associated with each symbol, namely: p(win) = 0.5 + pL + pR, where pL and pR are the adjustments associated with the symbol presented to the left and right of the screen. For instance, the symbols presented in the Figure 1A represent the pair of symbols A and Z (see Figure 1B) which were associated to the following probability of winning: p(win)AZ = chance + p(win)A + p(win)Z = 0.5 + 0.3 − 0.3 = 0.50. Optimal behavior in the task entailed betting eight points each time that a likely winning pair [i.e., p(win) > 0.5] appeared, and betting two points each time that a likely losing pair [i.e., p(win) < 0.5] was presented.

#### Cognitive reflection test (CRT)

The CRT (Frederick, 2005) is composed of three items that are designed to elicit an intuitive but incorrect response (“lures”) in face to a reasoning problem that requires a slow and reflexive response. The test includes the following questions:

- A bat and a ball cost $1.10 in total. The bat costs a dollar more than the ball. How much does the ball cost? _______ cents.
- If it takes 5 machines 5 min to make 5 widgets, how long would it take 100 machines to make 100 widgets? _______ min.
- In a lake, there is a patch of lily pads. Every day, the patch doubles in size. If it takes 48 days for the patch to cover the entire lake, how long would it take for the patch to cover half of the lake? _______ days.

A correct resolution of the test problems requires inhibiting or re-evaluating the first answer that pop up in the mind, to posteriorly reason and found the correct answer. In other words, it requires the inhibition of the so-called “system 1” (fast and intuitive) and the activation of the so-called “system 2” (slow and reflexive) (Evans, 2011). For example, the “bat and ball problem” is designed to elicit the intuitive answer “10” in conditions where the correct solution is “5.” The answers to (b) and (c) items are “5 min” and “47 days” respectively. In order to assess the performance in the CRT we used the same procedure in previous studies (Frederick, 2005; Oechssler et al., 2009). Specifically, we counted the number of correct responses and give one point for every correct answer. This procedure gave us a range of four points between 0 and 3. As in previous studies (Campitelli and Labollita, 2010; Hoppe and Kusterer, 2011), participants were instructed to answers the questions with paper and pencil without time restrictions.

### Behavioral data analysis

As already described, the probabilistic decision-making task that we used consisted of a series of trials where the subjects choose the amount of their bets (8 or 2) in response to a pair of cues that are associated with a specific probability of winning vs. losing. In the context of this task gain-learning can be defined as learning to detect trials with a probability of >50% of winning and bet high (i.e., 8) on those trials, whereas loss-learning can be defined as learning to detect trials with a probability of <50% of winning and bet low (i.e., 2) on those trials. In order to test if CRT scores predicts gain-learning and loss-learning, we pulled participants data from each trial to perform two separate logistic regression models. In the first model (Equation 1) we used CRT scores, gender and mathematical skills as predictors of choice behavior (i.e., betting high vs. betting low) in trials with a probability >50% of winning (i.e., gain-learning). In the second model (Equation 2) we used the same variables as predictors of choice behavior in trials with a probability <50% of winning (i.e., loss-learning). We introduced mathematical skills as a control for the ability to identify the correct answer in the CRT. This ability was measured using the score on the mathematical section of the National University Admission Test in Chile. Both the CRT and the score on the mathematical test were z-scored in order to facilitate their comparison. Thus, we evaluate the direct influence of cognitive impulsivity on choice behavior controlling for gender and mathematical skills as potential confounders.

(1) $$\begin{array}{l} \text{p }\left(\text{high}\;\text{bet}|\;\text{winning}\;\text{trial}\right)=\frac{1}{1+\;{\text{e}}^{-z}},\\ \text{ where}\;z\;=\;\beta_{0}+\beta_{\text{crt}}\;\times \;\text{CRT}+\beta_{\text{gen}}\times \;\text{gender}\\ \;+\;\beta_{\text{math}}\times \;\text{math}\;\text{skills}\;+\;\varepsilon \end{array}$$

(2) $$\begin{array}{l} \text{p}\left(\text{low}\;\text{bet}|\;\text{loosing}\;\text{trial}\;\right)=\frac{1}{1+\;{\text{e}}^{-z}},\\ \text{where}\;z\;=\;\beta_{0}+\beta_{crt}\;\times \;\text{CRT}+\;\beta_{\text{gen}}\times \;\text{gender}\;\\ \;+\;\beta_{\text{math}}\times \;\text{math}\;\text{skills}\;+\;\varepsilon \end{array}$$

## Results

### Behavioral results

Table 1 shows the percentage of subjects who correctly answer the CRT by the number of questions answered. Only 4.76% of participants correctly responded to the three items, whereas the 42.86% did not achieve any correct answer. The mean of correct answers was 0.95 (*SD* = 0.21). These results are within the range of previous studies. For example, in his original study, Frederick (2005) reports ranges that go from a mean of 0.57 correct answers in students from the Universidad de Toledo to a mean of 2.18 in students from the Massachusetts Institute of Technology. There was a significant effect for gender, *t*_(19)_ = 2.1877, *p* < 0.001, with males receiving higher scores (*M* = 1.44, *SD* = 1.01) than females (*M* = 0.58, *SD* = 0.79) on the CRT. This gender difference has also been found in previous studies, e.g., Frederick (2005), Oechssler et al. (2009), and Hoppe and Kusterer (2011).

**Table 1:** **Percentage of subjects whit correct answers by the number of items**.

**No. of correct answers**	**% Participants in the sample**
0	42.86
1	23.81
2	28.57
3	4.76

*n = 21*.

Interestingly, participants CRT's scores were not significatively correlated neither with reaction times on the task, *r*_(19)_ = 0.06, *p* = 0.78 nor with the number of no responses per participant, *r*_(19)_ = −0.20, *p* = 0.36, although there was an almost significant trend to correlate with the frequency of “too early” responses, *r*_(19)_ = −0.41, *p* = 0.06.

**Figure 2:**
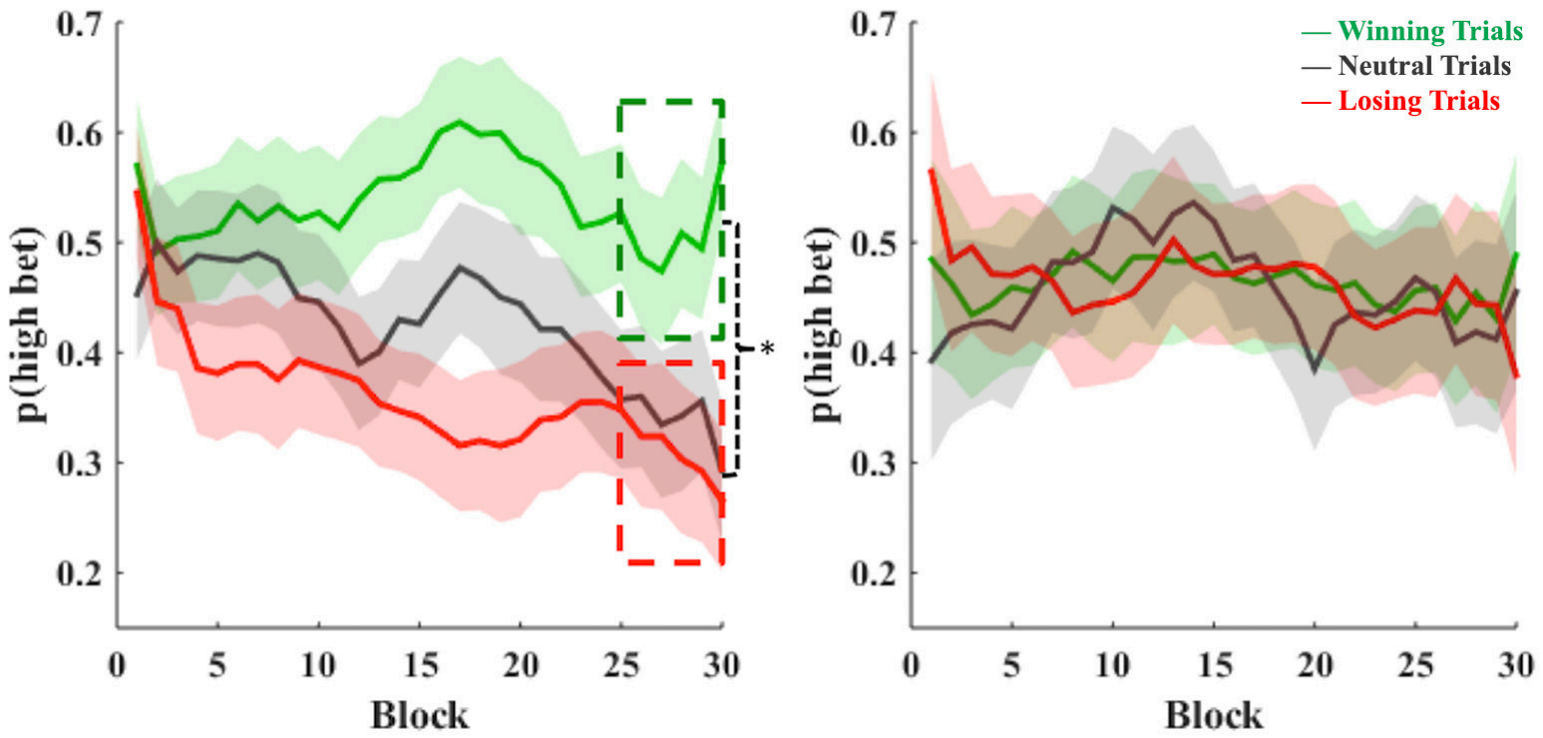
**Differences in choice behavior across blocks for low-impulsive subjects (left) and high-impulsive subjects (right)**. The vertical axis shows the choice behavior (betting high) for likely winning trials (green), neutral trials (gray), and likely losing trials (red) across the task. Every block represents 20 single trials in our gambling task. Shaded areas indicate SEM for each trace.

A visual inspection of choice behavior across blocks (Figure 2) suggests the existence of a difference in learning between high-impulsive participants, defined as the subjects who scored above the median of the distribution of the CRT scores, and low-impulsive participants, defined as the subjects who scored below the median of the distribution of the CRT scores. A *t*-test on the observed probability that low-impulsive participants would bet high on likely winning vs. the observed probability of doing so on likely losing trials found significant differences when we collapsed and analyzed the last five blocks of the task (i.e., blocks 26–30) (*M*_winning–*trials*_ = 51%, *SD*_winning–*trials*_ = 18%; *M*_losing–*trials*_ = 30%, *SD*_losing–*trials*_ = 21%; *t* = 2.57, *p* = 0.02), but not when we collapsed and analyzed the first five blocks of the task (*t* = 1.23, *p* = 0.22). We did not find significant differences when we repeated this procedure for high-impulsive participants (last five blocks, *t* = 0.12, *p* = 0.9; first five blocks, *t* = −0.85, *p* = 0.4). In summary only low-impulsive subjects, as measured with the CRT, learned to distinguish between likely winning and likely losing trials.

### Cognitive impulsivity predicts gain learning and loss learning

**Figure 3:**
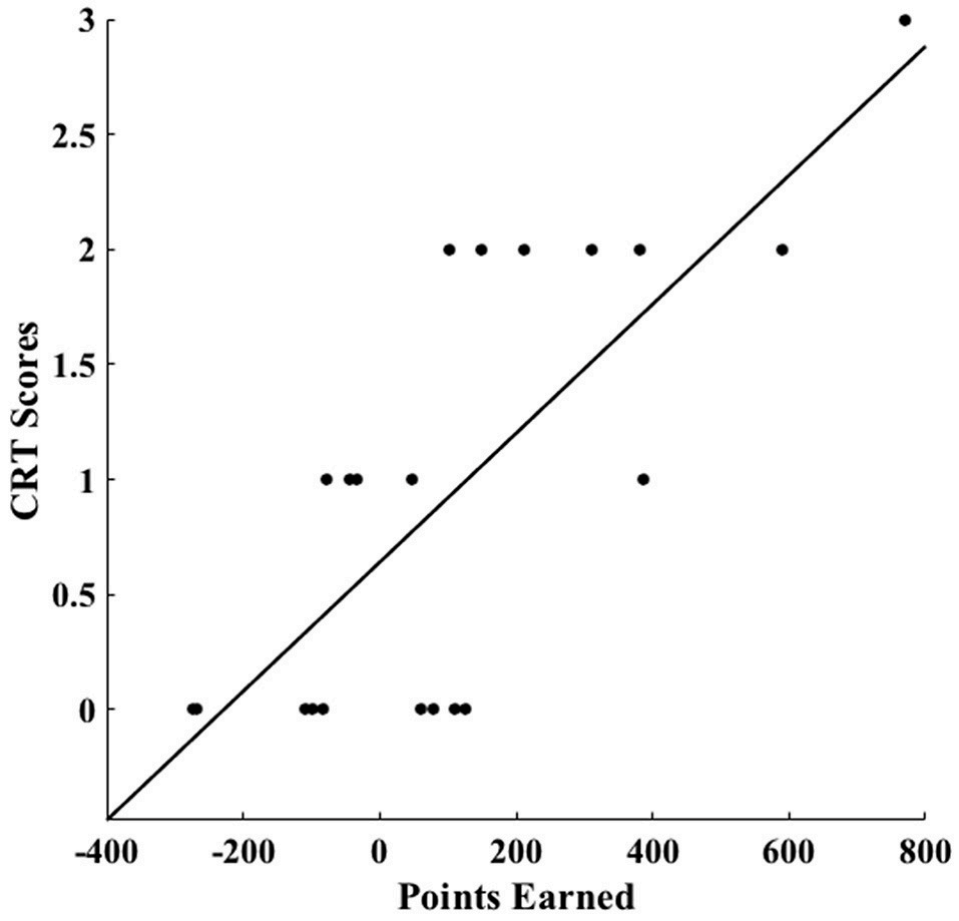
**Association between CRT scores and points earned during the task**. Across participants, lower cognitive impulsivity, as measured by the CRT, was associated with a better overall performance in our probabilistic decision-making task (*r* = 0.75, *p* = 0.001).

On average, participants collected 111 points during the task (*SD* = 262.1), with males achieving a mean of 197.3 points (*SD* = 260.83) and females a mean of 46.7 points (*SD* = 254.62); nevertheless such difference did not reached statistical significance [*t*_(19)_ = 0.017, *p* = 0.199]. Importantly, individual differences in this measure of overall performance scaled with CRT scores (*r* = 0.75, *p* = 0.001; Figure 3). In order to disentangle the specific contribution of gain-learning and loss-learning to this association, we performed two separated logistic regression models with CRT scores, gender, and mathematical skills as predictors of choice behavior (see Materials and Methods section). Detailed results can be observed in Table 2. Across participants, we found that CRT scores scaled both with the log odds for the tendency to bet high on likely winning trials (i.e., gain-learning) (*b* = 0.256; *p* = 0.000), and the log odds for the tendency to bet low on likely losing trials (i.e., loss-learning) (*b* = 0.409; *p* < 0.000). This was the case even after controlling for gender and mathematical skills.

**Table 2:** **Association between CRT scores and gain and loss learning**.

	**Choice behavior**					
	**Gain learning**			**Loss learning**		
**Model predictors**	**B**	**SE B**	***p***	**B**	**SE B**	***p***
Constant	−0.027	0.042	0.514	0.735	0.045	0.000
CRT	0.256	0.034	0.000	0.409	0.038	0.000
Gender	0.124	0.074	0.094	−0.696	0.078	0.000
Math	−0.109	0.032	0.001	0.267	0.035	0.000

## Discussion

Our results shed new light on the relationship between reward-based learning and cognitive impulsivity. Specifically, they show an association between the performance in the CRT, a measure of cognitive impulsivity, and the performance in a reward-based probabilistic decision making task. Our data analyses allowed us to disentangle the specific contribution of gain and loss learning to this association. The analysis showed, as we expected, that both loss and gain learning were positively associated with CRT scores, an association that remains significant even after controlling for mathematical skills and gender as potential confounders. This suggests that low cognitive impulsivity is related with better performance on both betting high on likely winning situations and betting low on likely losing situations.

Our study was motivated in part by previous research showing that both impulsivity and reward-based learning are associated with dopaminergic signaling (Frank et al., 2004; Kalivas and Volkow, 2005; Dalley et al., 2007; Klein et al., 2007; Schönberg et al., 2007; Pattij and Vanderschuren, 2008; Pizzagalli et al., 2008; Besson et al., 2009; Lee et al., 2009; Eagle et al., 2011; Eppinger et al., 2011; Jocham et al., 2014; Cox et al., 2015; Buckholtz et al., 2016). Our results supported the hypothesis of an association between individual differences in cognitive impulsivity and individual differences in learning-based choice behavior. These results contribute to the study of the interaction between these neurocognitive processes and decision-making. An unresolved question, however, is the causal directionality behind the associations that we reported here. Here we briefly discuss two potential explanations that are not necessarily mutually exclusive. One possibility is that individual differences in reward-based learning explain individual differences in cognitive impulsivity. This possibility is partially supported by studies on attention-deficit/hyperactivity disorder (ADHD) and Parkinson's disease, which have proposed that the heightened impulsivity that characterized these conditions is partially caused by an atypical oversensitivity to positive rewards compared to negative rewards (Frank et al., 2004, 2006, 2007; Williams and Dayan, 2005; Frank, 2006; Luman et al., 2009, 2010; Maia and Frank, 2011). Another possibility is that impulsivity precedes choice behavior, modulating the ability to learn the association between cues and winning/losing probabilities. According to this view, individual differences in cognitive control could explain individual differences in reward-learning. This second view is indirectly supported by findings on the influence of impulsivity on addiction and maladaptive decision-making patterns, which has pointed to impulsivity as a premorbid risk factor of addictive disorders and poor decision-making (Bechara, 2005; Dalley et al., 2007; Verdejo-García et al., 2008; MacKillop et al., 2011).

Finally, recent studies have started to shed light on the association between decision-making in the laboratory and real life outcomes (Ersner-Hershfield et al., 2009; Knutson et al., 2011; Kuhnen, 2015). For example, Knutson et al. (2011) have shown a positive correlation between gain learning and assets accumulation and a negative correlation between loss learning and debt accumulation. Several studies have shown that debt accumulation is also associated with high levels of impulsivity in decision-making (Attitude et al., 2006; Joireman et al., 2010; Ottaviani and Vandone, 2011; Gathergood, 2012; Mansfield et al., 2013; Achtziger et al., 2015), and that saving behavior and economic wealth is associated with high levels of self-control (i.e., low impulsivity) (Laibson et al., 1998; Mastrobuoni and Weinberg, 2009; Moffitt et al., 2011). An issue for future research will be to determine the causal directionality or meditational effects behind these associations. In the context of the aforementioned studies and the results that we reported here, an interesting direction for future studies could be to evaluate whether cognitive impulsivity acts as a mediator of the suggested relationship between reinforcement learning skills and financial life outcomes. Future research could also delve in these associations by looking at their neurocognitive underpinnings and by analyzing their behavioral implications in different decision-making domains.

## Author contributions

RS and PC designed research; PC performed research; RS and PC analyzed data; PC and RS wrote the paper.

## Funding

This work was supported by grant FONDECYT (1161715) to RS.

### Conflict of interest statement

The authors declare that the research was conducted in the absence of any commercial or financial relationships that could be construed as a potential conflict of interest.
